# Editorial: Geospatial and Transport Modeling in Stroke Service Planning

**DOI:** 10.3389/fneur.2019.01057

**Published:** 2019-10-01

**Authors:** Thanh G. Phan, Richard Beare, Noreen Kamal

**Affiliations:** ^1^Neurosciences, Monash Health, Melbourne, VIC, Australia; ^2^School of Clinical Sciences, Monash University, Melbourne, VIC, Australia; ^3^Central Clinical School, Monash University, Melbourne, VIC, Australia; ^4^Academic Unit, Peninsula Health, Frankston, VIC, Australia; ^5^Developmental Imaging, Murdoch Children's Research Institute, Melbourne, VIC, Australia; ^6^Department of Industrial Engineering, Dalhousie University, Halifax, NS, Canada

**Keywords:** geospatial, transport, model, access, stroke

Recent advances in acute stroke therapy, endovascular clot retrieval, and clot busting drugs (1, 2), have re-focused emphasis on appropriate and timely transportation of patients for treatment. The implementations of these medical breakthroughs are equally as important as the clinical trials and are critical to translation of these trial findings. The implementation phase requires support in terms of government policy (Allen et al.; Mathur et al.), pre-hospital (Holodinsky et al.; Phan et al.; Tajaddini et al.; Vidale et al.), and hospital cares (Seah et al.). At all stages, one needs to develop models with fair access for all patients to the best stroke therapy whether they live in metropolitan or remote areas (Mathur et al.). These desires need to be balanced by the limited workforce of neurologists with interest in stroke or stroke physicians.

Complementing these advances, exploration of ideas in operational research can help the balancing act in determining appropriate hospitals for transport given the degree of stroke severity for that patient. In this special issue, investigators had created a web based apps to allow clinicians to estimate the impact of various additions (e.g., adding CT Perfusion or Telemedicine) to mobile stroke unit (MSU) on the operating range of MSU (Phan et al.). These investigators found that the range of MSU can be extended to 76 min from base. On the other hand, addition of CT Perfusion and Telemedicine would reduce the operating range of MSU. Investigators next explored the location of MSU in a metropolitan city (Phan et al.). This study reveals that some inner city suburbs are well-serviced and within 30 min of up to 10 hospitals. One may surmise that the deployment of MSU in these over serviced suburbs may not be fair. Perhaps MSU should be deployed in the peripheral suburbs that are less well-served by hospitals providing either endovascular clot retrieval (ECR) or clot busting therapy (Phan et al.). In the exploration of the location for clot retrieval hubs, investigators found several different combinations of hospital which can serve a metropolitan city as Sydney, Australia (Phan et al.). The investigators extended their earlier work with mapping location of clot retrieval service in Melbourne (3), Australia by adding the constraint of expected number of clot retrieval cases. The expected number of cases can be estimated from the census data for each suburb and stroke incidence studies (2). The investigators found that assuming a 3-hospital model and 15% of patients eligible for clot retrieval, the expected number of cases to be handled by each hospital is 465 (~9 cases per week). This number drops down to 374 if a 4-hospital model is preferred (~7 cases per week) (Phan et al.).

Remote and sparsely populated areas and low income countries present very different challenges to delivery of emergency stroke services, with limited clinical resources, long travel times, and well-known disparities in many key metrics in comparison to urban populations. Improved care delivery involving MSU, including airborne MSU, is complex (Mathur et al.). Investigators have reviewed a range of strategies used by health services internationally when deploying MSU and explored the complex interplay between distribution of health-care resources, expertise, costs, pre-hospital screening, and technological advances, including telemedicine.

Pre-hospital screens are a key component in decision making by emergency services personnel, potentially contributing to the choice of the most appropriate treatment destination for the stroke patient. If, for example, occlusions of large arteries can be detected reliably (Holodinsky et al.; Phan et al.; Tajaddini et al.; Vidale et al.) in pre-hospital screens then patients can be transported directly to emergency clot retrieval centers, bypassing smaller centers and avoiding delays (Lima et al.). The research shows how these screening tools can be combined with technologies and models to assess which hospital the patient should be transported to Holodinsky et al. and Lima et al. Additionally, transport of stroke patients has often been dichotomized as two potential options: Drip and Ship, where the patient is first transported to the *closest* thrombolysis then transferred to the *closest* clot retrieval hospital; and Mothership where the closest stroke hospital is bypassed in favor of the *closest* clot retrieval hospital (Holodinsky et al.). However, a study shows that all potential transport options should be considered (Schlemm et al.).

Improvement in efficiency of communication between clinicians in the extremely complex stroke treatment pathway has the potential to reduce the time to reperfusion of stroke cases. An open source application (Seah et al.), based around secure mobile phone communication and web interfaces and linking management, assessment, and investigation teams is undergoing trials. The application exploits real-time geospatial information to track patients and arrival times of physicians. Investigators intend to make their software freely available. Continuing the theme of open source software underpinning improvements in stroke service delivery is an introduction to open source geospatial analysis tools (Padgham et al.). A tutorial-style introduction to fundamentals of geospatial analysis, in the context of stroke service delivery, is provided. Source code and data are available to support researchers becoming familiar with possibilities offered by the vast range of online data and visualization services and freely available analysis tools.

Mathematical models have also played a critical role in the creation of best pre-hospital transport destination as well the optimization of the location stroke centers. Mathematical algorithms that take into account drive times, hospital efficiencies, and pre-hospital stroke screening tools developed from the decay curves of pooled randomized control trial data have been used to create an online interactive software that shows best transport destination for the patient, and the usability of this software has been assessed (Holodinsky et al.). This software can be used by healthcare administrators to develop pre-hospital transport protocols that define transport boundaries based on hospital performance. Similarly, a multi-object genetic algorithm was developed to optimize the number hyper-acute stroke hospitals in England, which used the following optimization parameters: number of hospitals; travel time; proportion of patients within 30 min; and number of stroke admissions to the hospital (Allen et al.). This study was used to influence policy.

We often assume that ambulance travel time is much faster than that by car. This scenario may not be true (3) as some countries do not allow ambulance traveling with light and sirens to drive the wrong way down the road. This is worrying as the population in metropolitan areas increases with time and timely transport of patients will become a major issue (Tajaddini et al.). We had explored this issue by using strategic transport model and which contains data on proposed road network and future population growth. These models have an advantage over Google Maps application programming interface (API) as this latter technology based the trip time estimate on crowd source data on moving cars and current roads (not proposed roads in the future). A disadvantage of such strategic model is that the road network data used in the modeling process is deemed confidential as some proposed road may or may not be built.

The vision of this Research Topic in the Stroke section of Frontiers in Neurology was to discuss contemporary issues in pre-hospital care, emergency service transport of patients (including mobile stroke unit) and operational research into optimization of these transport models. It is hoped that the discussion can stimulate the readers to design a transport model to meet their local needs. Some of the papers in this special issue have source codes and data available for the adventurous readers to explore. Happy mapping and modeling.

## Author Contributions

All authors listed have made a substantial, direct and intellectual contribution to the work, and approved it for publication.

### Conflict of Interest

The authors declare that the research was conducted in the absence of any commercial or financial relationships that could be construed as a potential conflict of interest.
